# Potency Assays for Mesenchymal Stromal Cell Secretome-Based Products for Tissue Regeneration

**DOI:** 10.3390/ijms24119379

**Published:** 2023-05-27

**Authors:** Georgy Sagaradze, Anna Monakova, Anastasia Efimenko

**Affiliations:** 1 Institute for Regenerative Medicine, Medical Research and Education Center, Lomonosov Moscow State University, 27/10, Lomonosovskiy av., 119192 Moscow, Russiamonakovaao@my.msu.ru (A.M.); 2 Faculty of Medicine, Lomonosov Moscow State University, 27/1, Lomonosovskiy av., 119192 Moscow, Russia

**Keywords:** regenerative medicine, mesenchymal stem/stromal cells, conditioned medium, secretome, potency assay, quality control, translation, stem cell niche, mechanism of action, spermatogonial stem cell niche, male infertility

## Abstract

Adult stem cells maintaining tissue homeostasis and regeneration are tightly regulated by their specific microenvironments or stem cell niches. The dysfunction of niche components may alter the activity of stem cells and ultimately lead to intractable chronic or acute disorders. To overcome this dysfunction, niche-targeting regenerative medicine treatments such as gene, cell, and tissue therapy are actively investigated. Here, multipotent mesenchymal stromal cells (MSCs), and particularly their secretomes, are of high interest due to their potency to recover and reactivate damaged or lost stem cell niches. However, a workflow for the development of MSC secretome-based products is not fully covered by regulatory authorities, and and this issue significantly complicates their clinical translation and has possibly been expressed in a huge number of failed clinical trials. One of the most critical issues in this regard relates to the development of potency assays. In this review, guidelines for biologicals and cell therapies are considered to be applied for the development of potency assays for the MSC secretome-based products that aim for tissue regeneration. Specific attention is paid to their possible effects on stem cell niches and to a spermatogonial stem cell niche in particular.

## 1. Introduction

Stem cell niches containing stem and progenitor cells contribute to sustaining tissue renewal and regeneration in adult organisms [1]. If the components of a stem cell niche are damaged or not functioning properly, this could disrupt stem cell activity and lead to chronic or acute untreatable conditions [2,3]. To restore altered stem cell niches, regenerative medicine treatments such as gene, cell, and tissue therapy are actively investigated [4].

At present, cell therapy approaches with multipotent mesenchymal stromal cells (MSCs) as main actors are being actively studied as these cells can potently activate the body’s own resources to replenish damaged or lost niche components [5]. Although MSCs can differentiate into missing microenvironment components in order to endow a niche with the ability to regulate stem cell fates, they are involved in tissue regeneration primarily due to paracrine secretion. These cells are able to release cytokines, growth factors, components of the extracellular matrix, and extracellular vesicles (including nucleic acids in their composition), transfer organelles to target cells to restore damaged stem cell niches, and maintain stem cell pools during tissue recovery [6]. Commonly, MSCs have been used for bone, cardiac, neurodegenerative and immunological disorders and for traumas. There are more than 10 approved MSC products worldwide as well as more than 250 recruiting clinical trials to date [7,8,9]. However, many trials on MSC therapy have failed despite encouraging preclinical data and safety profiles [10]. These observations highlight the potential advantages of MSC secretome-based products, which exert the therapeutic functions of MSC and are devoid of many natural and technological drawbacks of cell therapy such as the unwanted differentiation of injected cells and the possible formation of tumors or ischemic disorders [11,12,13,14,15,16,17,18].

Nevertheless, the development of MSC secretome-based products still remains complicated [19,20,21]. Among many factors, one can select the development of potency assays as a substantial challenge. Therefore, in our review, specific attention has been paid to the potency assessment of MSC secretome-based products. Due to the lack of documents covering the development of potency assays for the MSC secretome, we have analyzed guidelines for biological drugs and cell therapies and underlined common patterns to follow. Given the requirement that potency assays should reflect the mechanisms of action, we considered stem cell niches and their distinct components as potential therapeutic targets of complex MSC secretome compositions, making them useful as appropriate tools for potency assay development [22]. Taking a representative example, we discussed in detail the contribution of MSCs to the regulation of a spermatogonial stem cell niche mediated by cell secretome to demonstrate the possibilities of utilizing this knowledge to test the potency of MSC secretome-based products for male infertility treatment.

## 2. MSC Secretome-Based Products: A Pharmaceutical Classification

Although the professional community is highlighting the regulatory challenges facing the pharmaceutical development of MSC-derived products, there are no guidelines fully covering their development to date [23,24]. Therefore, relying on documents regulating drugs similar to MSC secretome may provide additional benefit for their pharmaceutical development (Table 1).

MSC secretome can be considered a medical product as it is intended to treat, prevent, or diagnose a disease, or to restore, correct, or modify physiological functions, by exerting a pharmacological, immunological, or metabolic action [25,26]. Among medical products, MSC secretome is the closest to biologicals and cell therapeutics. However, irrespective of its biological nature, MSC secretome falls outside the definition of a biological product [27]. Similar to why they cannot be considered biological medicinal products by the U.S. Food and Drug Administration (FDA), most MSC-derived products cannot be considered biological medicinal products by the European Medicines Agency (EMA) as the guidelines of these entities can only be applied to products that can be highly purified and characterized using an appropriate set of analytical procedures [28]. Although ultrafiltration can be used for fractionation and purification, and mass spectrometry can serve as an analytical method for the composition of MSC secretome-based products, these methods cover only a limited set of products due to their complex nature and high donor-to-donor variability [29,30,31,32]. Conversely, MSC secretome can be classified as a biological drug by the Eurasian Economic Union (EAEU), as this entity’s list includes medicinal products produced using various biotechnological processes [33]. Hence, the prevailing position is that the MSC secretome cannot be developed based on guidelines for biological drugs alone.

Similar to what occurs with biologicals, the MSC secretome falls outside the scope of cell products and advanced therapy medicinal products (ATMPs) as the regulatory authorities require accepted products to consist of cells that are subject to substantial manipulations [34,35]. As exceptions, MSC exosomes carrying recombinant mRNAs can be considered ATMPs as their therapeutic effects relate directly to the recombinant nucleic acid sequences they contain [33,36].

Taken together, from a regulatory point of view, MSC secretome-derived products are neither biologicals nor cell products or ATMPs. However, due to their biological nature and the cellular origin of the secretome components, as well as common regulations in some authorities [34], the key requirements for both biological and cellular products will be considered together in order to formulate the best approaches to the potency assessment of MSC secretome-based products in regenerative medicine.

**Table 1:** Inconsistencies between the definitions of MSC secretome and various classes of medical products.

	The Eurasian Economic Union	The European Union			The United States of America	
Terms	Biological medicinal products	Medicinal products	Advanced therapy medicinal products (ATMP)	Biological medicinal products	Human cells, tissues, and cellular and tissue-based products (HCT’Ps)	Biological medicinal products
Reasons for qualifying	Contain a biological active substance that is produced by or extracted from a biological source and that needs, for its characterization and the determination of its quality, a combination of physicochemical-biological testing together with the production process and its control	Are intended to treat, prevent, or diagnose a disease, or to restore, correct, or modify physiological functions by exerting a pharmacological, immunological, or metabolic action				Include a wide range of products such as vaccines, blood and blood components, allergenics, somatic cells, gene therapy, tissues, and recombinant therapeutic proteins. Biologics can be composed of sugars, proteins, nucleic acids, or complex combinations of these substances, or may be living entities such as cells and tissues.
Reasons for not qualifying			ATMP should contain or consist either of cells or tissues that have been subject to substantial manipulation so that biological characteristics, physiological functions, or structural properties relevant for the intended clinical use have been altered, or of cells or tissues that are not intended to be used for the same essential function(s) in the recipient and donor.	Are produced from recombinant or non-recombinant cell-culture expression systems and can be highly purified and characterized using an appropriate set of analytical procedures.	Contain or consist of human cells or tissues that are intended for implantation, transplantation, infusion, or transfer into a human recipient. The HCT/Ps are minimally manipulated	Biological products mean a virus, therapeutic serum, toxin, antitoxin, vaccine, blood, blood component or derivative, allergenic product, protein, analogous product, or arsphenamine or derivative of arsphenamine (or any other trivalent organic arsenic compound) that is applicable to the prevention, treatment, or cure of a disease or condition in human beings.
References	[33]	[35]	[36,37]	[28]	[38]	[27,39]

## 3. Development of Potency Assays for MSC Secretome-Based Products for Tissue Regeneration

To suggest the best approaches to potency assessment, a review of the documentation governing the handling of biologicals and cell therapies was carried out. Potency tests are usually performed in vivo and/or in vitro and, less often, using physicochemical methods. In vivo tests can either be performed in an animal model mimicking the studied clinical effect or be based on a drug’s mode of action [40]. Simple as well as complex endpoints can be selected for representative animal models in order to assess the potencies of test products [41].

In vitro potency assays can be designed based on physiological responses under defined conditions as well as direct or surrogate correlates of the presumed biological activity. A potency assay should reflect or mimic the product’s known or intended mechanism of action. For example, consider vascular endothelial growth factor (VEGF), which was considered a surrogate marker of the angiogenic potency of an MSC-based product in a study. First, the authors of the study showed that VEGF secreted by MSCs functionally induced angiogenesis. Then the significant correlations between VEGF concentration in the MSC secretome and its in vitro effects, such as endothelial cell migration, proliferation, and vascular tube formation, were shown [42]. If the mechanisms of MSCs are mediated by multiple effector molecules, a matrix approach can be used. Based on a hypothesis that MSC-mediated increase in chemokine milieu affected multiple immune cells providing a therapeutic outcome due to functional immune suppression, chemokine secretion signature in MSCs and peripheral blood mononuclear cell cocultures was thoroughly explored in another study [43]. If a bioassay is not directly associated with the mechanism of action, it may be necessary to show a relationship between the selected assay and some other assay that better or otherwise reflects the mechanism of action of the test product [41]. If a variability in test systems is expected, an absolute measure of potency may not be available, and a relative potency methodology may be considered [44]. A standard can be established by regulatory authorities or internally [41]. For such a complex product as MSC secretome, characterization of a standard can be based on a panel of factors that are presented with a high degree of uniformity between analyzed samples [45].

To guarantee the quality of a potency assay, validation tests for specificity, precision, linearity, accuracy, and range should be carried out [24,46]. To fulfill the specificity criterion, the assay should be able to measure the analyte (the substance of interest) unequivocally in the presence of various impurities. For MSC secretome-derived products, impurities may include materials from the manufacturing steps, like cell culture supplements or preservatives, as well as the degradation products of cells or their secretomes [47]. For example, to show specificity, one can add samples with a known concentration to show that the assay results are unaffected by the presence of impurities or excipients [48]. Alternatively, the assay can be performed in complete medium and compared to the relevant results in the control solution [19].

Precision is usually expressed as the variance, standard deviation, or coefficient of variation of a series of measurements. It is sufficient to show intra-assay precision under the same operating conditions over a short interval of time along with intermediate within-laboratory precision, where the day of experimentation, equipment, or analysts may vary [49,50]. In the aforementioned study investigating VEGF as a surrogate factor for MSC potency, several batches of MSC secretome were measured by two operators and by a single operator on two different days. For both studies, the coefficients of variations, standard deviations, and results of *t*-tests were presented [42]. It is noteworthy that intermediate precision may be replaced with between-laboratory precision [49].

Linearity should be evaluated across the range of the analytical procedure. If there is a linear relationship in the data, test results should be evaluated by using appropriate statistical methods: for example, by the calculation of a regression line using the method of least squares. In some cases, to obtain linearity, the test data may need a mathematical transformation prior to regression analysis. The correlation coefficient, y-intercept, slope of the regression line, and residual sum of squares should be submitted along with the data plot. If the data transformation does not help achieve linearity, the analytical response should be described by an appropriate function of the concentration [49]. For example, to assess the linearity of the inhibitory potential of MSCs in T-cell proliferation, different MSC concentrations were tested while keeping the number of peripheral blood mononuclear cells constant. T-cell proliferation, the formation of daughter generations, and the inhibition of T-cell proliferation by MSCs were investigated [51]. For potency assays, a range of 80 to 120 percent of the expected test concentration should be minimally considered. A minimum of 5 concentrations is generally recommended in order to establish linearity [49].

Accuracy, the closeness of agreement between an accepted conventional true value or accepted reference value and the value found, should be established across the specified range of the analytical procedure [49]. The draft guidance on Bioanalytical Method Validation by the United States Food and Drug Administration defines accuracy in terms of “closeness of mean test results” [52]. In order to assess accuracy, the most common approach involves the construction of target potency via the dilution of reference standards or test materials with known potencies and then the calculation of relative bias at each individual target potency level [53]. For both active substances as well as medicinal products (if it is impossible to obtain samples of all product components), accuracy can be measured by using an alternative well-characterized procedure that incorporates stated and/or defined accuracy by adding known quantities of the analyte to the product [49]. In the aforementioned assessment of the inhibitory potential of MSCs on T-cell proliferation, the accuracy of the studied assay was measured compared to the well-defined test that had been used as the performance criterion of the MSC qualification [51]. It is noteworthy that for both active substances as well as medicinal products, accuracy may be inferred once precision, linearity, and specificity have been established [49].

Taken together, the key requirements for potency assays of biological and cellular products are rather consistent and may be used for the development of MSC secretome-based products.

## 4. Stem Cell Niche Recovery as a Promising Endpoint for Potency Assays of MSC Secretome-Based Products

Presumably, in response to injury, MSCs promote regeneration by preserving an adult stem cell pool and/or restoring their supportive microenvironment, or a stem cell niche, to ensure their subsequent appropriate functioning in damaged tissues [54,55]. To fulfill this important function, MSCs secrete extracellular matrix components (ECMs), growth factors, regulatory RNAs, and proteins within extracellular vesicles, and even transfer some organelles. Hence, as analytes for potency assays, one may consider evaluating the selected MSC secretome factors that possibly maintain stem cells and their niches [6,20].

In order to focus on stem cells as targets of MSC secretome-based products, individual factors that are necessary and sufficient for their maintenance and function may be selected as analytes for potency testing. For example, it was suggested that TGFBI, an ECM glycoprotein, regulated the function of the epidermal stem cells and promoted wound healing [56]. For intestinal epithelial repair after inflammation- or irradiation-induced injury, it was shown that MMP17, the membrane-bound matrix metalloproteinase, which is exclusively expressed by smooth muscle cells, is required [57].

**Figure 1:**
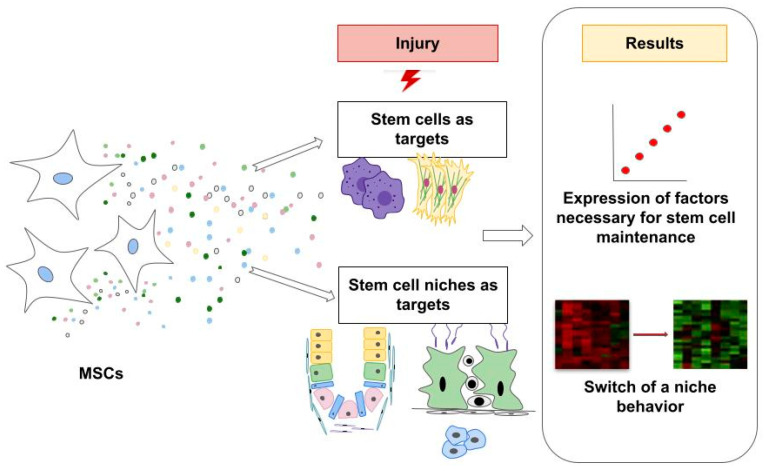
Both the expressions of factors necessary for stem cell maintenance as well as the indicators of a stem cell niche behavior switch are suggested analytes for the potency testing of MSC secretome-based products.

However, the identification of independent markers of the potency of MSC secretome largely relies on low throughput screens. Moreover, sometimes, the only factor sufficient for recovery may not be established. Additionally, given the effort required to establish individual metrics for each marker, functional attribute, or disease condition, it seems reasonable to develop assays applicable for a broad range of activities [58,59,60]. Hence, the restoration of a whole damaged stem cell niche may be worth considering for analysis in potency assays (Figure 1). To support tissue regeneration, some adult stem cell niches may switch to developmental-like niches after an injury [61]. Hence, it has been proposed that the niche environment instructs the transition of neural stem cells to and from dormancy [62]. It is noteworthy that the analysis of transition from activation to dormancy has revealed the list of upregulated genes shared with muscle, hematopoietic, and hair follicle epidermal stem cells, suggesting common behavior patterns for their niches [63].

Taken together, both the expressions of individual factors reflecting the maintenance of stem cells as well as whole niche changes directing stem cells towards regeneration may be selected for analysis in potency tests for MSC secretome-based products.

## 5. Spermatogonial Stem Cell Niche as a Promising Target to Assess Potency of MSC Secretome-Based Products for Idiopathic Male Infertility

Spermatogenesis is a process by which spermatogonial stem cells (SSCs) are self-renewed and differentiated into haploid male gametes. It proceeds in a complex testicular microenvironment organized as two structurally discrete compartments: seminiferous tubules and the surrounding interstitial space. A cross-section of seminiferous tubules shows a composite epithelium of somatic and germ cell types. In the periphery of these seminiferous tubules, there are spermatogonia and Sertoli cells [64]. The latter play a key role in spermatogenesis by maintaining spermatogonial populations as well as through the paracrine regulation of other testicular somatic cells [65]. The outer surface of the seminiferous tubules is covered by peritubular myoid and lymphatic endothelial cells. The space between the seminiferous tubules is filled with interstitial cells including testosterone-producing Leydig cells, nerve fibers, connective tissue cells, and various immune cell populations, as well as blood vessels that never penetrate the seminiferous tubules [66,67].

Accumulating evidence indicates the contribution of some testicular somatic components to the pathogenesis of idiopathic male infertility [68]. It is known that Sertoli cells can inhibit the mitogenic activity of Leydig cells, and that such dysregulation in testicular failure is often represented by Leydig cell hyperplasia [69,70]. The contribution of SSC niche cells may be also confirmed by single-cell RNA-seq analysis of the testicular microenvironment showing signs of fibrosis and inflammation in cases of infertility. In particular, cryptozoospermic patients have demonstrated a stronger interaction between blood vessel cells, immune cells, and undifferentiated spermatogonia through cytokine signaling [71,72]. Other components of the SSC niche, testicular macrophages, are involved in spermatogenesis, steroid production and immune privilege. In the absence of macrophages, defects in differentiation of undifferentiated spermatogonia as well as decrease in spermatogonial proliferation were observed. Probably, the disturbances in these interactions were associated with the declined secretion of CSF1 by macrophage that normally stimulated SSC self-renewal. Testicular macrophages also secrete 25-Hydroxycholesterol which can be converted into testosterone by Leydig cells, and produce factors that maintain testicular immune privilege. Due to indispensable role of these cells in the maintenance of spermatogenesis, their dysfunction may lead to pathological conditions including idiopathic male infertility [73,74,75,76,77].

Peritubular myoid cells secrete glial cell line-derived neurotrophic factor (GDNF), which is required for the self-renewal of SSCs [78]. Although GDNF is also secreted by Sertoli cells, GDNF derived from peritubular myoid cells is shown to be essential to maintain the populations of undifferentiated spermatogonia in mice [79].

These findings are highly relevant as infertility affects about 15–20% of couples worldwide and a male factor is involved in about half of the cases, of which 30–50% are idiopathic with no effective treatment options [80,81]. Although commonly used approaches such as assisted reproductive technologies and empirical medical treatments including hormonal therapy, antioxidants, and selective estrogen receptor modulators may improve sperm parameters such as sperm concentration, motility, and morphology, or even increase pregnancy rates, the results of randomized clinical trials or systematic reviews disprove these conclusions [82,83,84,85]. Thus, the effective treatment of idiopathic male infertility remains an unmet medical need, and targeting the somatic testicular microenvironment with MSC-based products may be a particularly promising strategy [54,86,87]. MSCs have been shown to be located in the testicular interstitium contacting Leydig cells, macrophages, and cells within the blood vessel wall. Recently, using a rat cryptorchidism model, we demonstrated that both MSCs and the components of MSC secretome could efficiently stimulate spermatogenesis and the production of functional germ cells, leading to the restoration of Sertoli cell pools and Leydig cell secretory functions, thus supporting the recovery of SSC niches [55]. However, similar to what has been noted in other applications, here we face the challenges involved in the development of such substances, including the development of potency assays, which should reflect the possible role of MSC-SSC niche interactions in the pathogenesis of idiopathic male infertility.

Many effects of MSC secretome on SSC niches are currently known. In the murine model of busulfan-induced spermatogenesis injury, it was shown that the systemic administration of MSC secretome leads to the increased mRNA expression of N-cadherin and ICAM1, which are involved in the barrier function of Sertoli cells. Moreover, the MSC secretome-treated group in the study showed the best results in experimental wound healing by the TM4 Sertoli cell line in a scratch assay conducted in the early stages of observation [88]. This result was consistent with the hypothesis that MSC secretome could attract Sertoli cells to the injured niche from specific transient zones of the adult testes. Although the results were statistically insignificant due to a low number of replicates, MSC secretome had stimulated Sertoli cell migration [89,90]. Given that the functional indicators of Sertoli cells are observed along with increases in spermatogonia populations, the expressions of genes or proteins involved in the function of Sertoli cells, as well as their migratory potentials, can be suggested as endpoints for potency assay.

Leydig cells are considered essential for spermatogenesis and fertility due to their secretory function. An increase in serum testosterone concentration was demonstrated following local MSC secretome injections in a model of rat abdominal cryptorchidism [55]. It is noteworthy that the correlation between relative serum testosterone concentration and the total and moving fraction of spermatozoa was also shown in the study [90]. Taken together, androgen secretions by Leydig cells may be suggested as the endpoints of a potency assay to study MSC secretome-based products for their impact on male infertility. This may be supported by the ability of the MSC secretome components, at least of VEGF, to stimulate testosterone secretion by Leydig cells in vitro. However, the necessity and sufficiency of VEGF being the main stimulator of testosterone secretion by Leydig cells has not yet been reliably shown [20,91].

Taken together, the dysfunction of cells within SSC niches can be associated with idiopathic male infertility which has been demonstrated at the preclinical and clinical levels. Moreover, some cells have been found to be necessary for physiological spermatogenesis, and more such findings can be revealed in future [92]. At the same time, data regarding the effects of MSC secretome components on the niche-related mechanisms of male infertility are accumulating, supporting the translation of novel biological products that utilize the regenerative potency of MSCs in the field of male infertility treatment. However, to develop a potency assay for MSC secretome-based products, the effects of MSC secretome on the recovery of damaged SSC niche components essential for spermatogenesis may be considered. It is noteworthy that the studied pathogenetic mechanism should be shown both in humans as well as in selected model objects [93].

## 6. Concluding Remarks

MSC secretome-based products can satisfy many unmet medical needs in regenerative medicine. However, their development and clinical translation are complicated by the lack of clear workflows for such products. In particular, it is worth noting that there are no specific guidelines for the potency analysis of MSC secretome-based products. To bypass this issue, we analyzed the guidelines covering the development of biologicals and cell products and suggested common patterns to be followed. We have not revealed any specific requirements that are possibly applicable to MSC secretome.

Similar to what is required for biologicals and cell therapies, potency assays for MSC secretome-based products are required to reflect their mechanisms of action. Due to the presumption of the central role of MSCs as stem cell niche modulators, we highlighted the value of the established mechanisms of MSC contribution to niche regulation for the development of relevant and affordable potency assays for such products. Specifically, we have suggested to analyze individual factors within MSC secretome that are necessary for the regeneration of injured tissue or niche-wide changes switching the behavior of stem cells towards regeneration in potency testing. The advantages of the suggested approach have been demonstrated by the examples of some current MSC- and MSC secretome-based products which have been in development for various clinical indications.

## Data Availability

Data is available on request.
